# The novel trends and sustainable developments of graphene nanostructures for medical applications

**DOI:** 10.1039/d6ra06351a

**Published:** 2026-09-03

**Authors:** Sameer A. Awad, Ahmed A. Al-Khubaisi, Numan F. AlHeety, Eman M. Khalaf, Doaa A. Elsayed

**Affiliations:** a Department of Medical Laboratories Techniques, College of Health and Medical Technologies, University of Al Maarif Al Anbar Iraq sameer.msc1981@gmail.com; b Department of Forensic Evidence, College of Science, University of Al Maarif Al Anbar Iraq; c Department of General Science, College of Basic Education-Haditha, University of Anbar Al Anbar Iraq; d Department of Chemistry, College of Education for Women, University of Anbar Ramadi Iraq; e Department of Chemistry, Faculty of Science, Zagazig University Zagazig 44519 Egypt doaaatef641995@gmail.com doaaatef@zu.edu.eg +20-01207053013

## Abstract

Graphene nanoparticles (GNPs) have emerged as transformative nanostructures in biomedical science due to their unique physicochemical properties, including high surface area, exceptional mechanical strength, tunable conductivity, and versatile surface chemistry. Their multifunctional characteristics render them promising for applications in drug delivery, biosensing, tissue engineering, neural interfaces, and antimicrobial therapies. This review highlights recent advances in graphene-based nanostructures for medical applications, with a particular focus on sustainable developments and translational challenges. We also emphasise toxicity and biocompatibility considerations, discuss current limitations in clinical translation, and provide future perspectives. This work critically examines the latest sustainable developments in graphene nanostructures for medical use, underscoring the hurdles in clinical translation and outlining future directions for safer and more effective implementation.

## Abstract 2

Graphene nanoparticles (GNPs) are promising nanostructures for biomedical applications due to their unique physicochemical properties, including high surface area, mechanical strength, tunable conductivity, and versatile surface chemistry.

## Graphical abstract

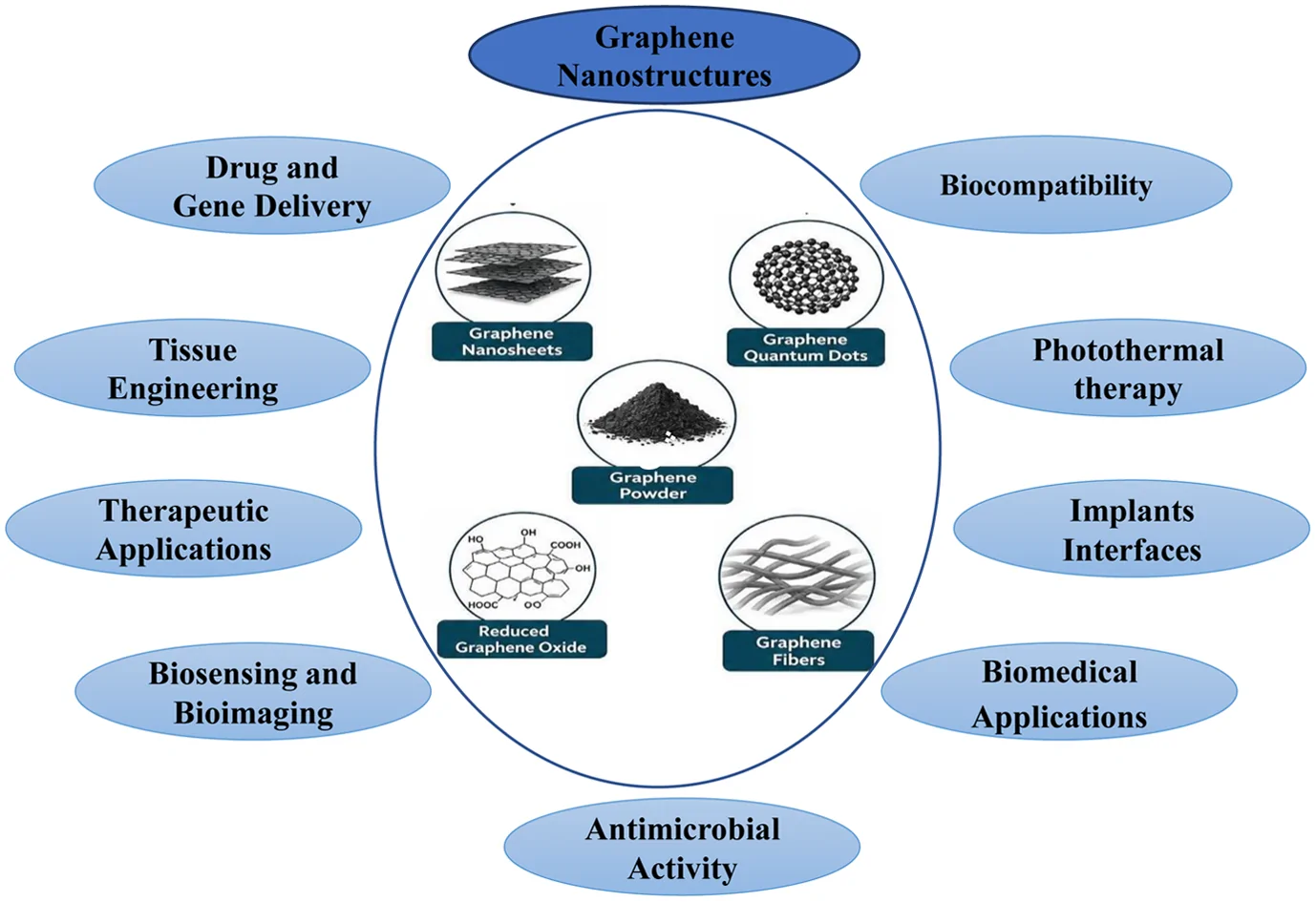

## 1 Graphene nanoparticles

**Fig. 1:**
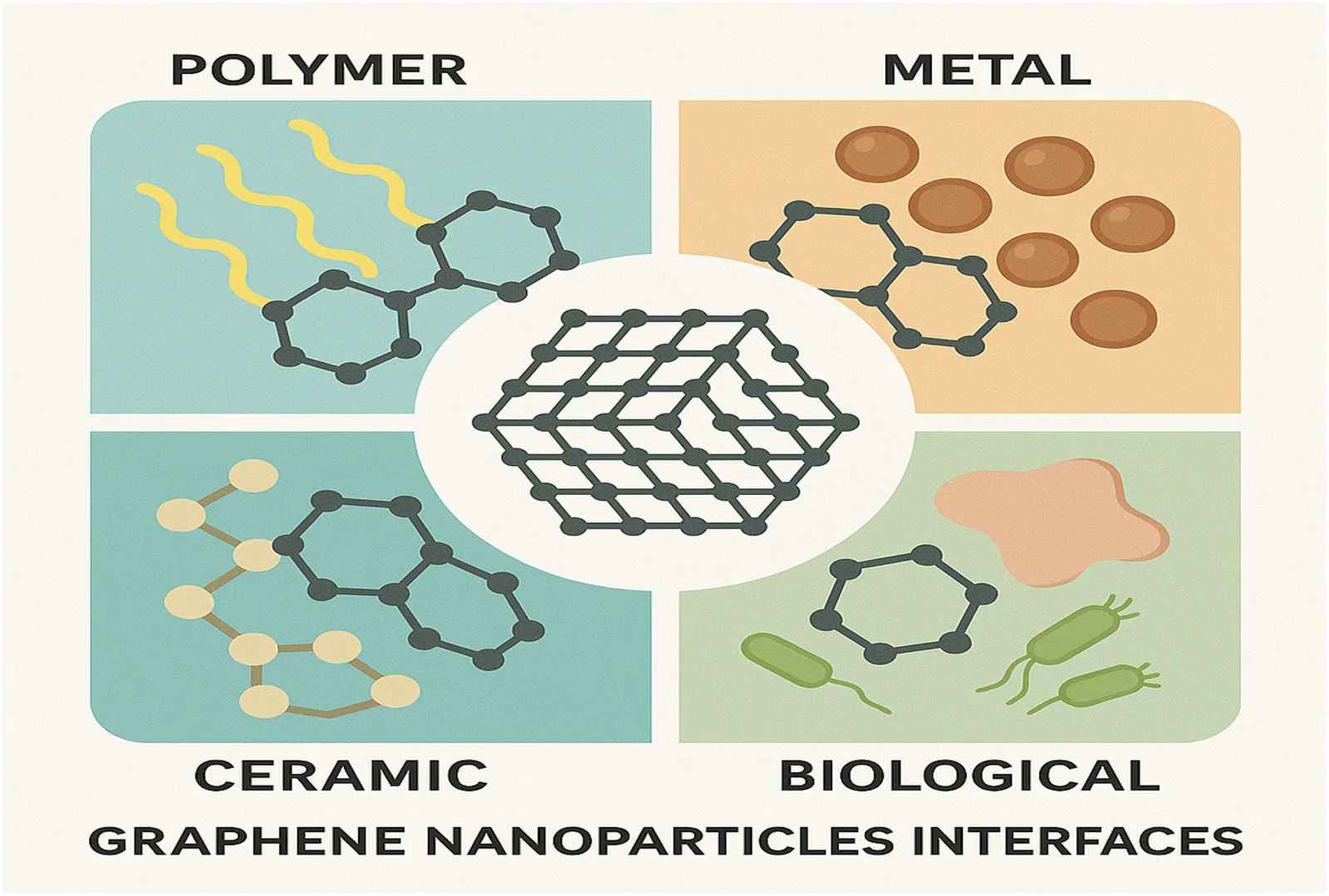
Graphene nanoparticle interfaces.

Graphene nanoparticles are nanoscale derivatives of graphene, typically with lateral dimensions below 100 nm.^1–6^ They can be synthesised by methods such as chemical vapour deposition, liquid-phase exfoliation, oxidation–reduction strategies, and thermal annealing. Since its isolation from graphite in 2004, graphene has revolutionised materials science, with applications extending from electronics and energy storage to aerospace, healthcare, and environmental remediation. Advanced characterisation tools, including atomic force microscopy (AFM), transmission electron microscopy (TEM), and Raman spectroscopy, have been pivotal in revealing its unique physicochemical properties and guiding the rational design of functional nanostructures.^7–12^ Structurally, graphene consists of a hexagonal honeycomb lattice of sp^2^-bonded carbon atoms. Its nanoscale modifications enable simultaneous hydrophobic and hydrophilic interactions: while certain functional groups, such as hydroxyl and carboxyl, render it hydrophilic and chemically reactive, pristine graphene surfaces remain largely hydrophobic and highly conductive. This duality renders graphene nanoparticles particularly suitable for biomedical applications such as drug delivery, wherein hydrophilic regions enable dispersibility in biological media, while hydrophobic domains facilitate the loading of poorly soluble drugs.^13,14^Fig. 1 illustrates the graphene nanoparticle interfaces.

The nanoscale form of graphene enhances the surface-to-volume ratio, reactivity, and bio-interfacing capacity. GNPs retain graphene's mechanical strength, electrical conductivity, and thermal stability, yet their reduced dimensions allow improved dispersion and surface functionalization.^15^ In electronics, they improve sensor sensitivity and battery charge storage. In medicine, they enable targeted drug delivery and imaging, while in environmental fields, they support pollutant adsorption and water purification.^16–18^ This versatility underpins the enormous potential of GNPs in driving sustainable technological innovations across multiple sectors. This review critically evaluates novel trends and sustainable developments in graphene nanostructures for medicine, examining their physicochemical basis, therapeutic versatility, biocompatibility, and the challenges that must be addressed for clinical translation.^19,20^

### 1.1 Comparative structure, property, biological relationships of graphene-based materials

Graphene-based materials are not a uniform group; their biomedical performance depends strongly on structure, surface chemistry, oxidation state, size, dispersibility, and biological interactions. Pristine graphene has a highly conductive sp^2^ carbon network but is hydrophobic and prone to aggregation, limiting its direct use in physiological environments. GO contains oxygen-rich functional groups that improve water dispersibility and enable drug loading or further functionalization, although its biological effects depend on size, oxidation level, aggregation, and dose. rGO partially restores graphene-like conductivity and NIR absorption but becomes less hydrophilic and more aggregation-prone, making stabilising surface modification important. Overall, defect density, surface chemistry, and the degree of conjugated network restoration are key determinants of graphene behaviour.^21–24^ Graphene quantum dots (GQDs) differ from larger graphene sheets because their nanoscale dimensions produce quantum confinement and edge effects.^25,26^ Typically, only a few layers thick and often below 10–20 nm, they exhibit tunable photoluminescence, size-dependent electronic properties, good aqueous dispersibility after functionalization, and efficient cellular uptake. These features make them especially useful for fluorescence imaging, biosensing, and theranostic applications.^22,27–30^ Their biological behaviour also varies by material type. Well-dispersed GO is generally more susceptible to enzymatic oxidative degradation because its oxygenated defects and hydrophilic surface promote enzyme interaction, while aggregated GO degrades less efficiently. Pristine graphene and poorly dispersed rGO may persist longer unless oxidised or functionalized. GQDs may show better dispersibility and possible clearance due to their small size, but their long-term fate remains dependent on formulation and surface chemistry.^31–35^ Toxicity is similarly formulation dependent. Hydrophobic graphene and rGO may interact strongly with cell membranes and aggregate, whereas GO can cause oxidative, inflammatory, or membrane-related effects depending on exposure conditions.^36^ Biocompatible coatings such as PEG, polysaccharides, or proteins can improve stability and reduce nonspecific interactions, but they may also alter uptake and biodistribution. Therefore, each graphene-based formulation must be evaluated individually rather than assuming that findings for one material apply to all graphene derivatives. The comparative physicochemical and biomedical characteristics of pristine graphene, GO, rGO, GQDs, and functionalized graphene systems are summarised in Table 1.

**Table 1:** Comparative physicochemical and biomedical characteristics of graphene-based materials

Property	Pristine graphene	GO	rGO	GQDs	Functionalized systems
Size/thickness	Up to 100 µm; ∼0.34 nm per layer	∼10 nm–µm; ∼0.7–1.2 nm per layer	∼10 nm–µm; ∼0.4–1 nm per layer	Usually <10–20 nm; few-layer	Formulation-dependent
Surface chemistry	Low oxidation; mainly sp^2^ C	Highly oxidized; –OH, epoxy, C=O, –COOH	Partially reduced; restored sp^2^ domains	Edge-rich –COOH/–OH; variable oxidation	Tunable by polymers, ligands, metals, *etc.*
Conductivity/dispersibility	Very high/poor	Very low/high	Moderate–high/low–moderate	Semiconducting/high	Tunable
Optical properties	Broadband absorption	Broad absorption; weak fluorescence	Strong NIR absorption	Strong tunable photoluminescence	Application-dependent
Biological behaviour	Aggregation/membrane interaction concerns	Biodegradable under some conditions; toxicity size/dose-dependent	Aggregation and hydrophobicity concerns	Often favourable uptake and relatively low acute toxicity	Improved stability, targeting and biocompatibility possible
Main biomedical value	Conductive interfaces	Drug loading/delivery	Photothermal therapy	Imaging, sensing, theranostics	Targeted/stimuli-responsive therapy
Ref.	37–40	38–42	37–40	40, 43 and 44	40–42

## 2 Biocompatibility and toxicity of GNPs

**Fig. 2:**
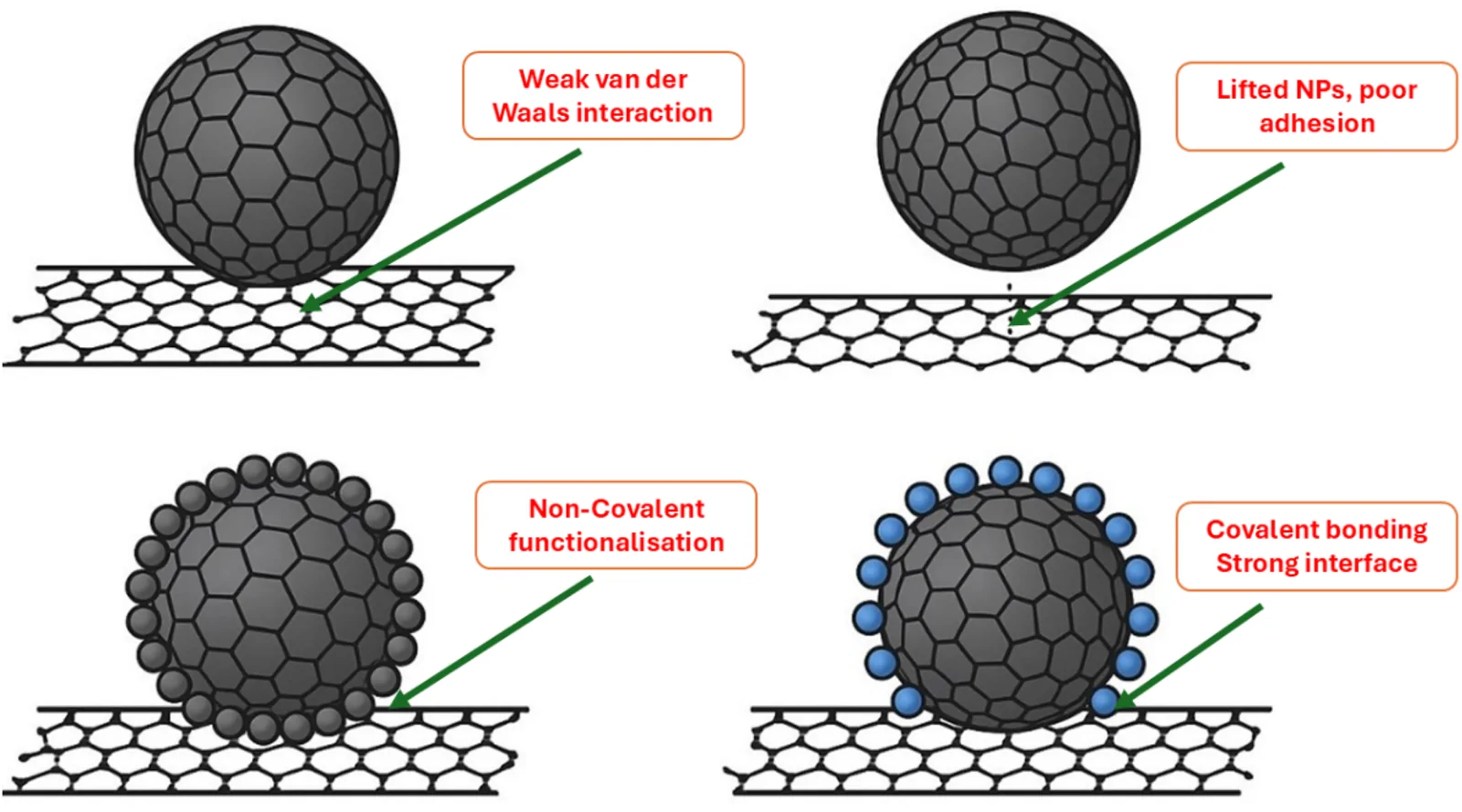
Mechanisms of graphene nanotoxicity and mitigation strategies through functionalization.

Graphene nanoparticles (GNPs), including graphene oxide (GO), reduced graphene oxide (rGO), and pristine graphene, have demonstrated substantial potential in biomedical applications such as drug delivery, biosensing, and tissue engineering due to their remarkable mechanical, electrical, and chemical properties.^42,45–49^ Nevertheless, understanding their interaction with biological systems remains critical. Several studies have shown that GNPs may display good compatibility under certain conditions; however, concerns persist regarding cytotoxicity, particularly in relation to particle size, surface chemistry, functionalization, and dosage.^50–52^ Toxicological studies indicate that graphene can induce the generation of reactive oxygen species (ROS), mitochondrial damage, membrane disruption, and inflammatory signalling.^53–55^ In addition, biodistribution and clearance pathways remain incompletely elucidated, creating uncertainty about long-term accumulation and chronic health effects.^56^ To mitigate toxicity, functionalization approaches such as PEGylation, chitosan conjugation, and hyaluronic acid coating have been applied. These modifications improve aqueous dispersibility, enhance colloidal stability, and reduce immune recognition. Functionalization also prolongs systemic circulation and promotes targeted uptake, collectively reducing cytotoxicity.^57^Fig. 2 illustrates the mechanisms underlying graphene nanotoxicity and mitigation strategies *via* functionalization.

## 3 Translational challenges and roadmap for graphene-based biomedical systems

Despite their strong potential, graphene-based biomedical systems still face major barriers to clinical translation, including inconsistent material standardisation, uncertain long-term biological behaviour, variable toxicity, manufacturing constraints, and unclear regulatory pathways. Progress will involve rigorous physicochemical characterisation, formulation-specific pharmacokinetic and biodistribution studies, evaluation of degradation and clearance mechanisms, long-term immunotoxicity and chronic toxicity assessments, reproducible GMP-compatible manufacturing, and robust regulatory-quality controls.^58–61^ Graphene-based platforms should therefore be evaluated as complete therapeutic products rather than as interchangeable graphenic materials, and current evidence should be described as preclinical unless supported by genuine clinical efficacy data.^62–64^ Standardised physicochemical characterisation is particularly important because biological behaviour is strongly influenced by size, layer number, thickness, oxidation level, defect density, surface charge, aggregation, contaminants, and functionalisation chemistry.^65–69^ Translational studies should use complementary techniques, including AFM/TEM, Raman spectroscopy, XPS, DLS, zeta-potential analysis, and contaminant testing, and should assess materials in biologically relevant media where protein adsorption and aggregation can alter their effective identity.^67,70^ Without harmonised characterisation, comparisons between studies remain difficult, and toxicity or efficacy findings may appear inconsistent.^68,71^ A second critical requirement is formulation-specific assessment of pharmacokinetics, biodistribution, biodegradation, clearance, and long-term safety. Graphene-based materials should not be assumed to behave similarly, because circulation time, organ accumulation, elimination, degradation, and immune interactions depend strongly on size, surface chemistry, dose, aggregation state, functionalisation, and administration route.^68,70–72^ Evidence from GO and PEGylated graphene studies shows distinct tissue distribution and persistence patterns, while enzymatic degradation by MPO or neutrophils depends largely on dispersion and material properties.^73,74^ Translational studies should therefore quantify degradation rates, identify degradation products, assess their toxicity, and establish mass balance and clearance over clinically relevant periods.^68,74^ Safety evaluation must also extend beyond acute cell-viability assays to include chronic immunotoxicity, inflammation, complement activation, oxidative and genotoxic stress, haematological effects, fibrosis, organ pathology, and repeated-dose toxicity. In parallel, future studies should define critical quality attributes, including size distribution, thickness, C/O ratio, surface charge, contaminants, endotoxin levels, functionalisation density, drug loading, release profile, and sterility, and confirm batch-to-batch reproducibility before regulatory progression.^68,75–77^

Clinical translation also depends on moving graphene-based biomedical systems from small-scale laboratory preparation to reproducible, GMP-compatible manufacturing. Many current platforms use complex functionalisation, lengthy purification, sonication, or ligand-conjugation steps that may be difficult to scale reliably and cost-effectively. Future development should therefore prioritise validated raw materials, controlled synthesis and purification, in-process quality monitoring, reproducible surface modification, validated analytical methods, sterilisation, stability testing, and defined product-release specifications.^58,78,79^ Overall, successful translation will require standardised material characterisation, long-term pharmacokinetic and toxicological assessment, scalable manufacturing, and regulatory frameworks that evaluate both the graphene material and the final therapeutic formulation. Current evidence for graphene-based biomedical platforms should be presented according to their true translational stage.^58,79,80^ Most drug-delivery, imaging, biosensing, and photothermal applications remain limited to *in vitro* or small-animal studies, which provide valuable preclinical proof of concept but do not demonstrate clinical efficacy. Although a recent randomised study reported that brief inhalation exposure to highly purified thin GO nanosheets was generally well tolerated in healthy volunteers, this was a human safety/exposure assessment rather than evidence of therapeutic benefit. Therefore, graphene-based drug-delivery and anticancer systems should not yet be described as clinically applied treatments,^40,58,81^Table 2.

**Table 2:** Translational status, available evidence, and remaining challenges for representative graphene-based biomedical systems

System/application	Current evidence	Translational stage	Main remaining requirements	References
GO drug-delivery systems	High drug loading, pH-responsive controlled release and extensive cellular studies	Predominantly *in vitro*	PK, biodistribution, chronic toxicity, standardised formulation	82–84
Targeted GO/DOX systems	Receptor-mediated targeting and tumour inhibition demonstrated in mice	Animal/preclinical proof-of-concept	Repeated-dose safety, PK/ADME, reproducibility, GMP manufacture	85
rGO chemo-photothermal systems	NIR photothermal activity, triggered drug delivery and anticancer efficacy	*In vitro* to animal/preclinical	Thermal dosimetry, chronic safety, clearance and scalable manufacture	86–88
GQD drug/theranostic systems	Drug loading/release, fluorescence and efficient cellular uptake; more limited animal therapeutic evidence	Predominantly *in vitro*/early preclinical	Long-term biodistribution, degradation, PK and therapeutic efficacy	89
PEGylated graphene/GO	Long-term biodistribution, pharmacokinetics and toxicology evaluated in mice	Preclinical safety evaluation	Standardised repeated-dose/GLP studies and regulatory-grade characterisation	71
GO/graphene biodegradation systems	MPO- and neutrophil-mediated degradation demonstrated experimentally	Mechanistic/preclinical safety evidence	Quantitative *in vivo* degradation, metabolite identification and clearance	41, 74 and 90
GO human inhalation exposure	Controlled 2 h exposure evaluated in 14 healthy volunteers	Human safety/exposure evidence—not therapeutic efficacy	Repeated/long-term exposure and application-specific clinical evaluation	81
Graphene therapeutic drug carriers overall	Extensive *in vitro* and animal evidence but no established clinical therapeutic efficacy	Predominantly preclinical	GLP toxicology, GMP manufacture, regulatory authorisation and therapeutic clinical trials	58, 66 and 68

### 3.1 Biocompatibility of GNPs

**Fig. 3:**
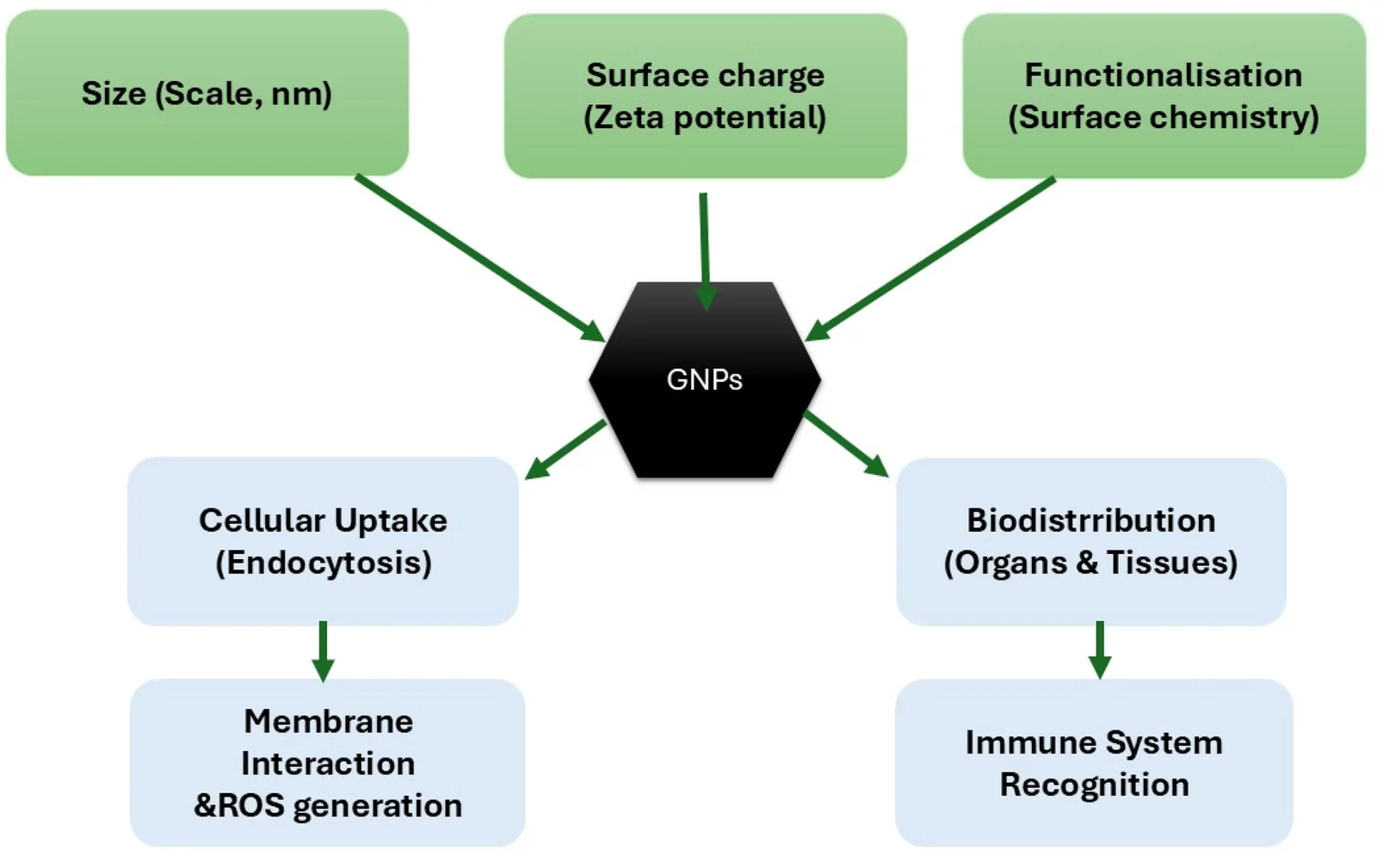
Key physicochemical factors (size, charge, functionalization) governing the biocompatibility of GNPs *in vitro* and *in vivo*.

The biocompatibility of GNPs is strongly dependent on surface chemistry, morphology, and physicochemical properties. Strategies like PEGylation, carboxylation, and hydroxylation improve circulation stability by increasing dispersibility and reducing protein adsorption.^13,91–94^ Functionalized graphene materials show superior compatibility with mammalian cells compared to pristine graphene, which tends to aggregate and interact nonspecifically. Cellular uptake mechanisms include clathrin-mediated endocytosis (CME), phagocytosis, and passive diffusion, enabling internalisation by macrophages, epithelial cells, and stem cells.^14,94–98^ At controlled concentrations, GNPs have been shown to support cell adhesion, proliferation, and differentiation, particularly when integrated into scaffolds for tissue engineering.^99^ Graphene-coated scaffolds promote osteogenic and neuronal differentiation, demonstrating applications in bone regeneration and neuroregenerative medicine.^100–102^ Biocompatibility outcomes are highly context-dependent: graphene quantum dots typically exhibit greater dispersibility and lower cytotoxicity, whereas larger graphene oxide sheets can induce oxidative stress and membrane damage.^58,60,68,103–105^ Surface functional groups (–COOH, –OH, –NH_2_) also influence protein corona formation, immune response, and biodistribution^68^Fig. 3.

### 3.2 Applications of GNPs' biocompatibility

**Fig. 4:**
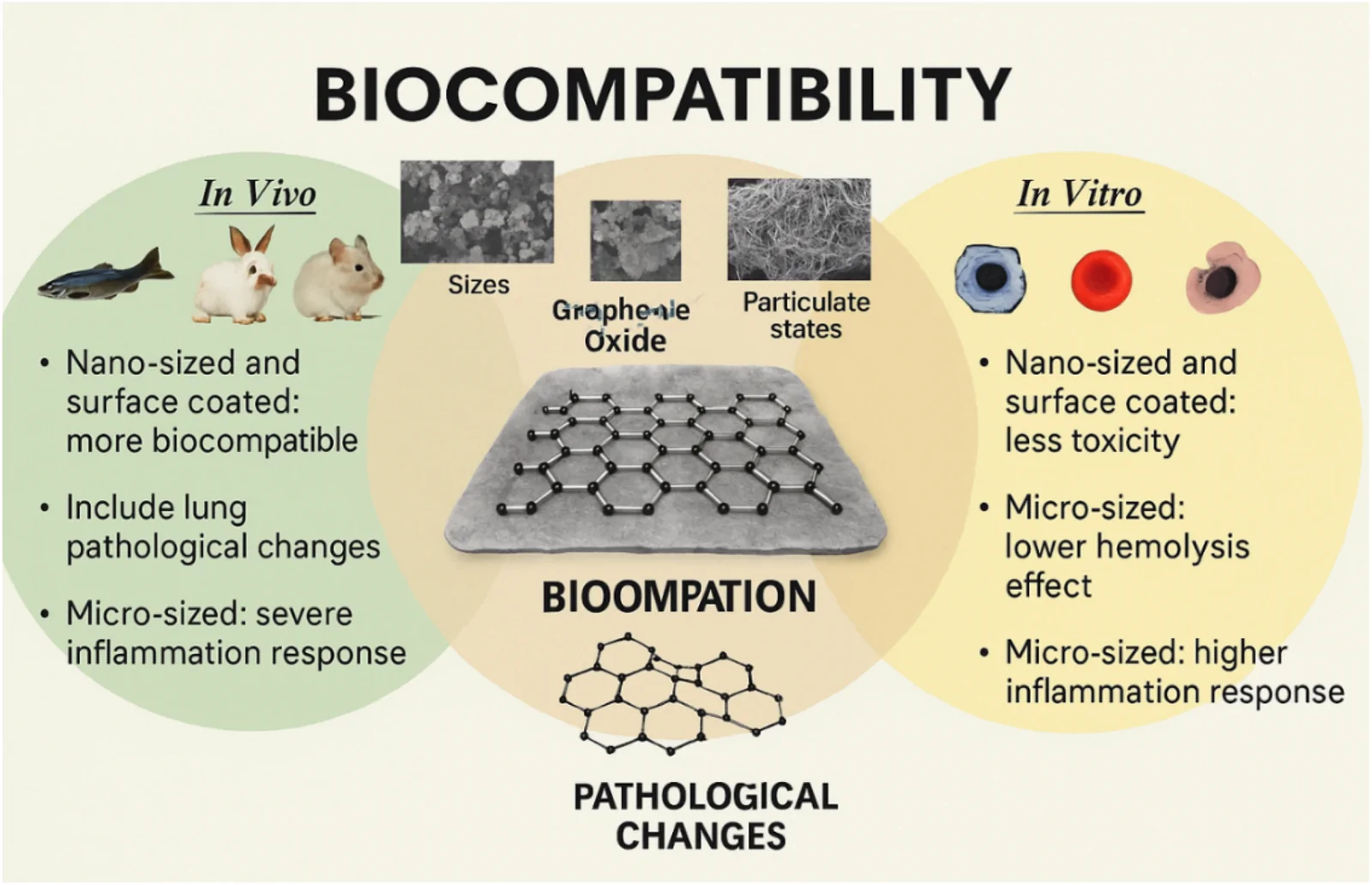
Key material physicochemical characteristics influencing the biocompatibility of the considered graphene-based materials in nanoforms.^79^

The biocompatibility of graphene nanoparticles (GNPs) has enabled their widespread use across various biomedical fields, particularly where safe interaction with cells and tissues is essential.^79,106–108^ In drug delivery systems, GNPs serve as efficient carriers due to their large surface area and π–π interactions, allowing high loading of therapeutic agents and controlled release profiles while minimising cytotoxicity.^58,99,106,109–111^ In tissue engineering, biocompatible GNP-based scaffolds are often combined with polymers such as chitosan or polyvinyl alcohol, which enhance cell adhesion, proliferation, and differentiation, making them suitable for bone, skin, and neural tissue regeneration.^13,112^ Additionally, GNPs are widely explored in biosensing applications, where their excellent electrical conductivity and compatibility with biomolecules enable sensitive detection of proteins, DNA, and disease biomarkers.^60^ In antimicrobial coatings, especially for medical devices and surfaces, functionalized graphene exhibits strong antibacterial activity while maintaining compatibility with human cells, reducing infection risks.^68,113–120^ Furthermore, in cancer therapy, GNPs are utilised in photothermal and photodynamic treatments due to their ability to efficiently convert light into heat while remaining relatively safe in biological environments when properly engineered.^121–125^ These diverse applications highlight that the controlled biocompatibility of GNPs is a key factor in advancing their clinical and technological potential,^68,126^Fig. 4.

## 4 Biomedical applications of GNPs

**Fig. 5:**
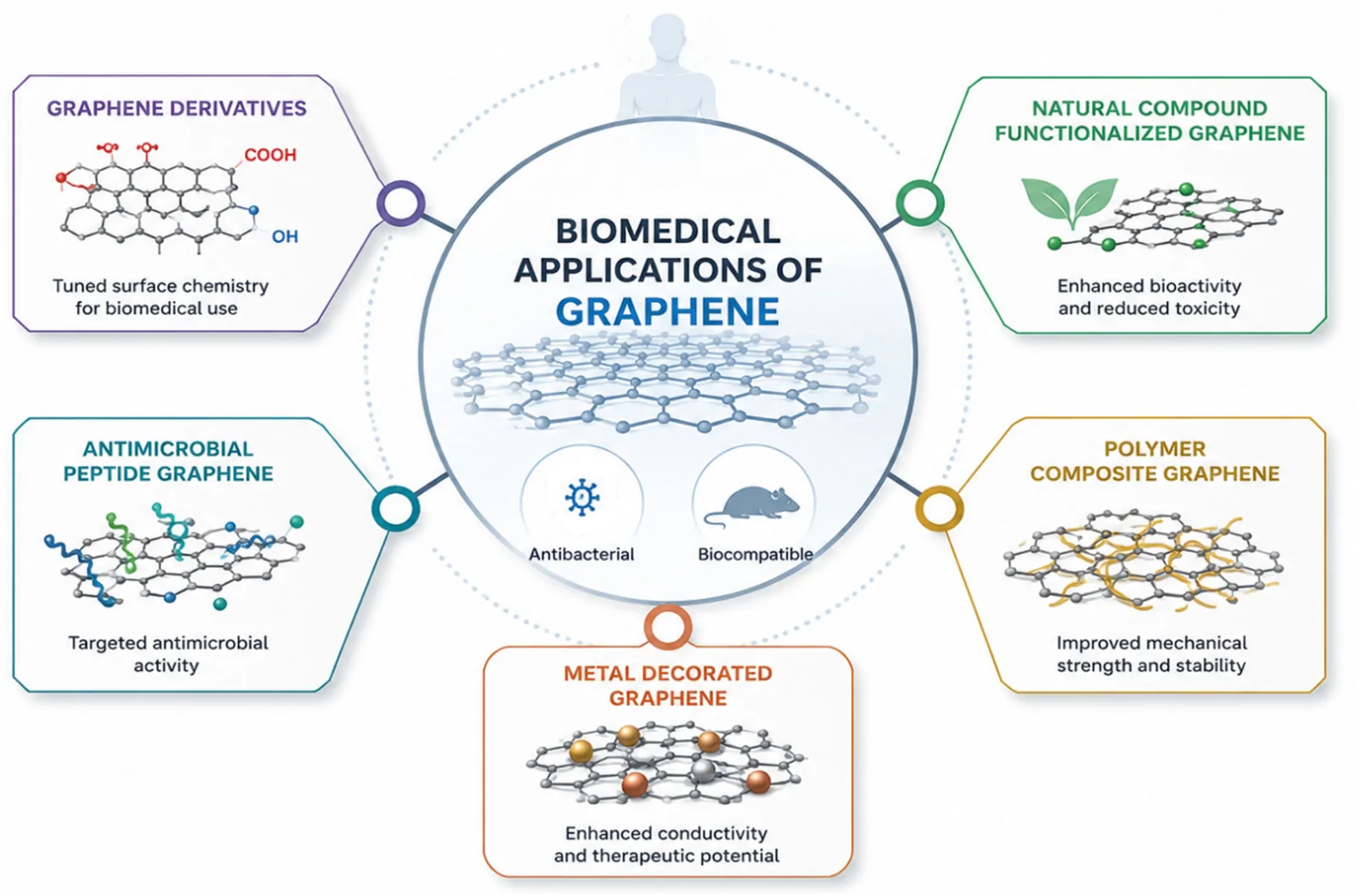
Types of graphene (GNPs) modifications discussed in this review for potential use in the biomedical field.^139^

Graphene oxide (GO) and reduced graphene oxide (rGO) serve as versatile platforms that can be functionalised with antimicrobial agents, natural compounds such as usnic acid, polymers, and metal nanoparticles, including silver, to enhance their biological performance.^116,127–131^ Such modifications significantly improve their compatibility with biological systems and expand their utility in areas including drug delivery, antimicrobial coatings, tissue engineering, and biosensing. The unique properties of graphene-based nanomaterials, such as tunable surface chemistry, high surface area, and strong interactions with biomolecules, make them highly suitable for advanced medical and therapeutic applications.^132–138^Fig. 5 shows the types of GNPs modifications discussed in this review for potential use in the biomedical field.

## 5 Drug and gene delivery

**Fig. 6:**
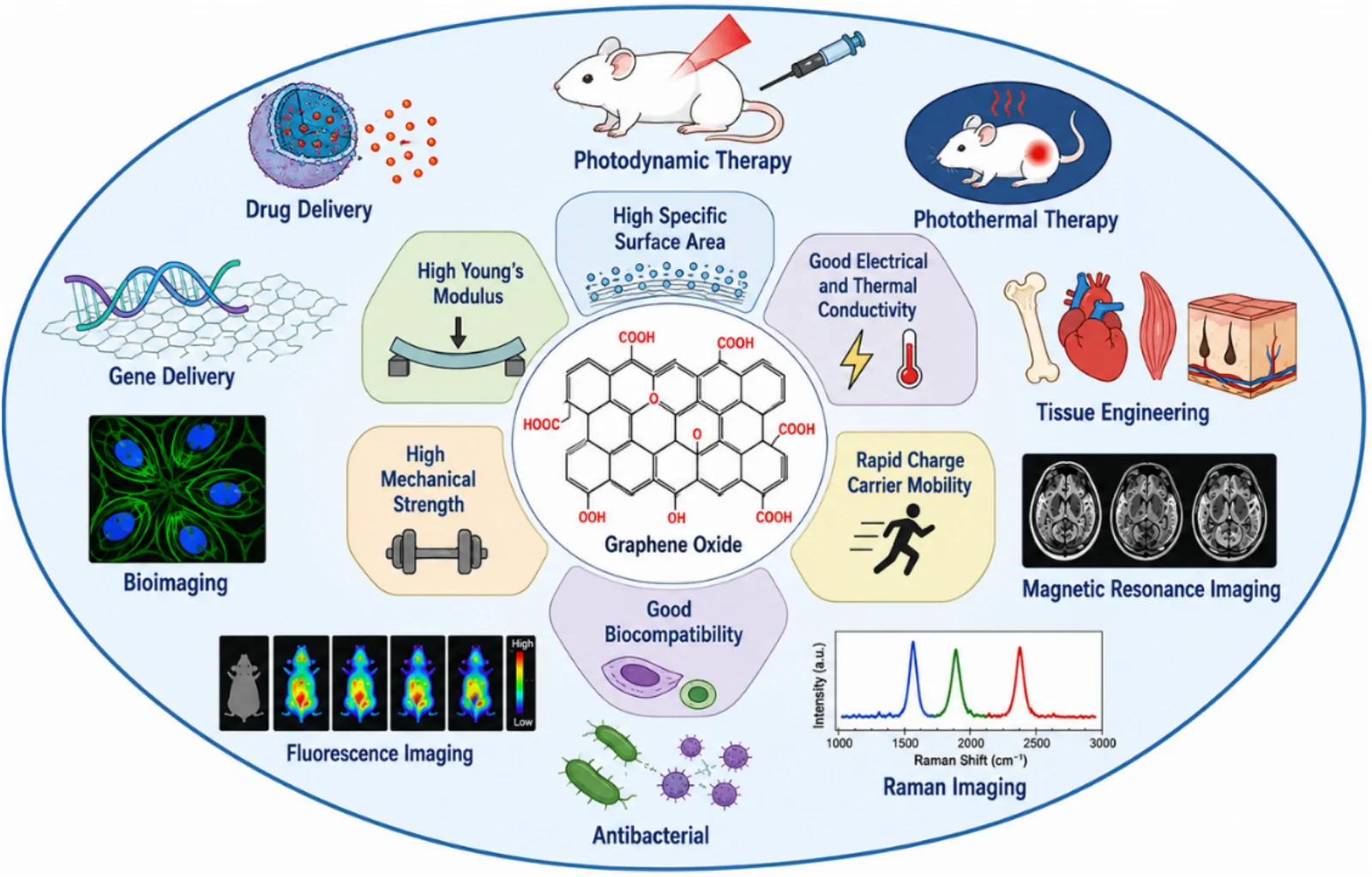
Shows the graphene-based nanocarriers for multifunctional drugs.

Graphene nanoparticles (GNPs) and their derivatives, including graphene oxide (GO), reduced graphene oxide (rGO), and graphene quantum dots (GQDs), have been extensively investigated as versatile drug and gene delivery platforms.^140–142^ Their exceptionally high surface area enables efficient drug loading, while π–π stacking and hydrogen bonding facilitate stable adsorption of therapeutic molecules and controlled release at target sites.^143–146^ Functionalization strategies, such as PEGylation or ligand conjugation, improve solubility, prolong circulation time, and enable tumour-specific targeting. For instance, doxorubicin-loaded GOs or GQDs have demonstrated enhanced anticancer efficacy by increasing tumour accumulation while reducing systemic toxicity.^143,147,148^ Previous research highlights the development of GO-based nano constructs for multimodal applications, including chemo-photothermal therapy, chemo-photodynamic therapy, bioimaging, and theragnosis in oncology. Collectively, these advances underscore the therapeutic potential of graphene nanomedicines, while also revealing challenges in clinical translation related to safety, reproducibility, and large-scale production,^149–151^Fig. 6 and Table 3.

**Table 3:** Advanced graphene-based nanoplatforms for cancer theranostics: diagnostic functions, therapeutic applications, and biomedical advantages

Graphene nanoplatform	Examples	Diagnostic role	Therapeutic role	Main advantages	References
Graphene oxide (GO)	GO, PEGylated GO, functionalized GO	Fluorescence, MRI/photoacoustic imaging after probe loading	Drug delivery, photothermal therapy, photodynamic therapy	High surface area, easy functionalization, strong drug adsorption	113
Reduced graphene oxide (rGO)	rGO, PEG-rGO, rGO-drug systems	Photoacoustic and NIR-based imaging	Strong photothermal therapy and chemo-photothermal therapy	Higher NIR absorption and photothermal conversion than GO	113
Graphene quantum dots (GQDs)	GQDs, doped GQDs, drug-loaded GQDs	Fluorescence bioimaging, tumour detection	Photodynamic therapy, photothermal therapy, and drug delivery	Photoluminescence, small size, good biocompatibility	152
GO–drug conjugates	GO–DOX, GO–paclitaxel, GO–cisplatin	Imaging-guided drug tracking	Chemotherapy with pH-responsive release	π–π stacking, hydrogen bonding, controlled release	153
GO–polymer hybrids	GO–PEG, GO–chitosan, GO–PLGA, GO–hyaluronic acid	Fluorescence/MRI, when combined with probes	Targeted drug/gene delivery and combination therapy	Improved stability, biocompatibility, and targeting	154
GO–metal nanoparticle hybrids	GO–Au, GO–Ag, GO–SPION, GO–Au–SPIO	MRI, CT, photoacoustic, multimodal imaging	Photothermal therapy, radio sensitisation, chemo-photothermal therapy	Combines graphene loading capacity with metal imaging/thermal effects	155
Photosensitizer-loaded GO/GQDs	GO–porphyrin, GO–chlorin e6, GQD-based PDT systems	Fluorescence-guided PDT	ROS-mediated photodynamic therapy	Enhanced photosensitizer delivery and tumour selectivity	156

### 5.1 Comparative drug-delivery performance of GO, rGO, GQDs, and functionalized graphene systems

Graphene-based drug carriers differ mainly according to oxidation state, sheet size, surface chemistry, and functionalization. Graphene oxide (GO) is widely used because its aromatic domains support π–π stacking with aromatic drugs, while its hydroxyl, epoxy, and carboxyl groups add hydrogen-bonding and electrostatic interactions. These features enable high drug loading, including reported DOX capacities in the mg mg^−1^ range; PEG/cetuximab-functionalized magnetic GO reached about 6.35 mg mg^−1^ and released more DOX at pH 5.5 than at pH 7.4, confirming tumour-relevant pH responsiveness.^157^ Other GO systems achieved about 85% DOX loading with faster acidic release.^158^ However, loading values should be compared cautiously because they depend on drug concentration, carrier/drug ratio, surface area, functionalization, and the way loading is reported. Reduced graphene oxide (rGO) has fewer oxygen groups than GO, so it offers weaker hydrogen-bonding and electrostatic interactions but stronger π-conjugation, hydrophobic adsorption, and near-infrared photothermal conversion. This makes rGO especially useful for chemo-photothermal therapy. For example, dopamine/Au-functionalized rGO loaded about 0.852 mg mg^−1^ DOX and released around 67% at pH 5.4.^158^ Hyaluronic-acid/cholesteryl-modified rGO also improved stability and CD44-mediated uptake, reducing tumour weight to about 14% of untreated controls in mice.^88^ Thus, rGO is valuable for aromatic drug adsorption and photothermal treatment, but it often requires PEG, polysaccharide, lipid, or protein functionalization to improve dispersibility. Graphene quantum dots (GQDs) are much smaller, usually only a few nanometres, which can reduce absolute drug payload compared with GO sheets. Nevertheless, their good aqueous dispersibility, intrinsic fluorescence, rapid cellular uptake, and possible clearance routes make them attractive theranostic carriers. One comparison showed GO reaching about 2.85 mg mg^−1^ DOX loading, while selected GQDs released up to about 78.1%, showing a trade-off between maximum loading and rapid release.^159^ Biotin-functionalized GQD–DOX also improved uptake in A549 cells and showed delayed nuclear accumulation after acidic intracellular drug release.^160^ Still, most GQD evidence remains *in vitro*, so tumour-targeting claims should not be directly extended to whole-body performance.

Surface functionalization usually provides the greatest improvement in biological performance. PEGylation enhances stability and circulation compatibility, while folate, peptides, antibodies, hyaluronic acid, and similar ligands support receptor-mediated targeting. Stimuli-responsive linkers such as hydrazone, disulfide, or protonatable groups can enable pH-, redox-, or dual-triggered release. For instance, PEGylated nano-GO with folic acid and hydrazone bonds showed faster acidic DOX release and better tumour-cell targeting,^161^ while disulfide-linked GO prodrugs were designed to respond to high tumour-cell glutathione levels. When combined with NIR irradiation, GO or rGO can also provide photothermal heating that accelerates release, increases membrane permeability, and damages tumour cells. Therefore, the improved efficacy of functionalized systems reflects the combined effects of graphene, drug loading, targeting ligands, responsive linkers, PEGylation, and photothermal action.^162^

For targeting, passive tumour accumulation through the enhanced permeability and retention effect should be separated from active molecular targeting. Ligand-functionalized systems provide stronger evidence when receptor-blocking or competition experiments confirm receptor-mediated uptake. For example, cetuximab-functionalized magnetic GO showed higher internalisation in EGFR-overexpressing CT-26 cells, and this uptake decreased after cetuximab pre-incubation. In mice, combining targeted GO/DOX with magnetic guidance and NIR irradiation reduced relative tumour volume from about 12.1 in controls to 0.42 by day 14, offering stronger therapeutic evidence than uptake data alone.^157^

Only a limited portion of graphene drug-delivery research has advanced from *in vitro* studies to meaningful *in vivo* testing. Cyclic-RGD/chitosan-functionalized GO carrying DOX produced nearly threefold greater tumour accumulation and a 56.64% tumour inhibition rate, about 2.9 times higher than free DOX, in tumour-bearing mice.^163^ Folate-targeted graphene systems achieved about 76% antitumour efficiency in 4T1 mouse models,^164^ and polymer-modified GO with DOX plus photothermal therapy reduced primary tumour growth and lung metastasis.^165^ These results indicate real preclinical promise, but they remain animal-model findings rather than proof of clinical translation, Table 4.

**Table 4:** Comparative drug-delivery performance and translational status of graphene-based nanomaterials^82–85,87–89,164–167^

Platform	Drug-loading characteristics	Release/response	Uptake & targeting	Evidence level
GO	Often highest; up to multi-mg drug/mg carrier	Strong pH responsiveness	Good after ligand modification	Extensive *in vitro*; several mouse studies
rGO	Strong aromatic-drug adsorption	pH + excellent NIR/thermal response	Requires stabilization/functionalization	Several promising *in vivo* chemo-PTT studies
GQDs	Usually, lower absolute payload than GO	Rapid pH-dependent release	Excellent uptake; intrinsic fluorescence	Predominantly *in vitro*; fewer robust *in vivo* studies
Functionalized GO/rGO/GQDs	Variable; surface coatings may increase or reduce accessible loading area	pH, redox, thermal or multi-stimulus responsive	Highest active-targeting potential	Best preclinical outcomes, but increased complexity

## 6 Biosensing and bioimaging

**Fig. 7:**
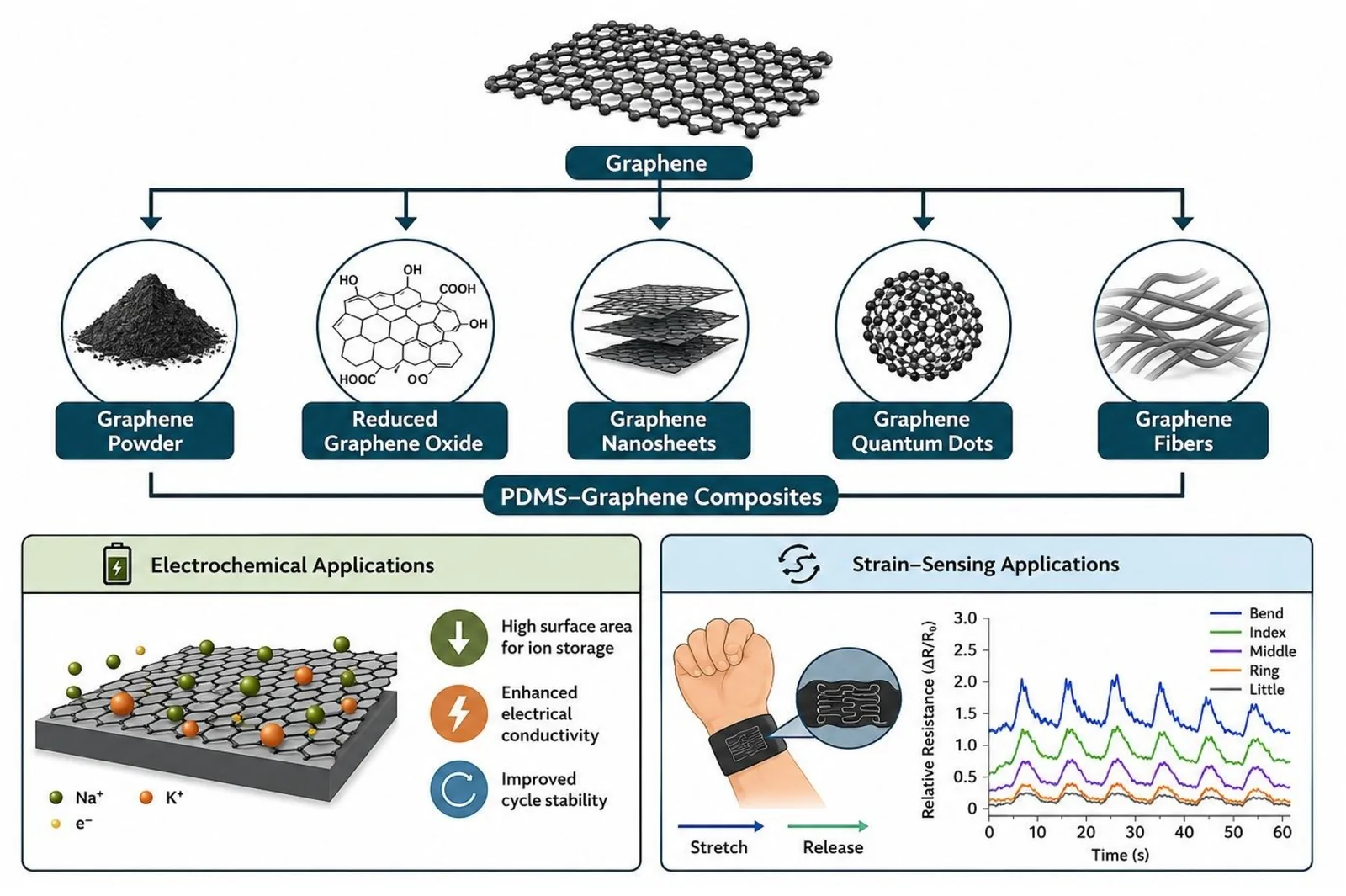
Schematic representation of graphene-based biosensing and bioimaging applications.

Graphene nanomaterials provide highly sensitive and versatile platforms for biosensing and bioimaging due to their exceptional conductivity, high surface-to-volume ratio, and strong fluorescence quenching properties.^168–173^ Functionalized GO nanosheets and GQDs have been applied in the detection of DNA, proteins, and cellular metabolites with high specificity and sensitivity.^174–178^ Their unique optical and electronic properties enable integration into electrochemical and optical biosensors, thereby significantly improving detection limits. In imaging applications, graphene-based nanoparticles enhance the contrast of multiple modalities, including fluorescence, magnetic resonance imaging (MRI), and photoacoustic imaging.^170,179,180^ Such multifunctionality enables precise disease diagnosis and real-time monitoring of therapeutic interventions, positioning graphene nanomaterials as promising candidates for next-generation diagnostic platforms. Graphene oxide (GO) and reduced graphene oxide (rGO) act as versatile platforms that can be functionalised with antimicrobial agents, natural compounds such as usnic acid, polymers, and metal nanoparticles like silver to enhance their biological performance,^181,182^Fig. 7.

## 7 Tissue engineering and therapeutic applications

**Fig. 8:**
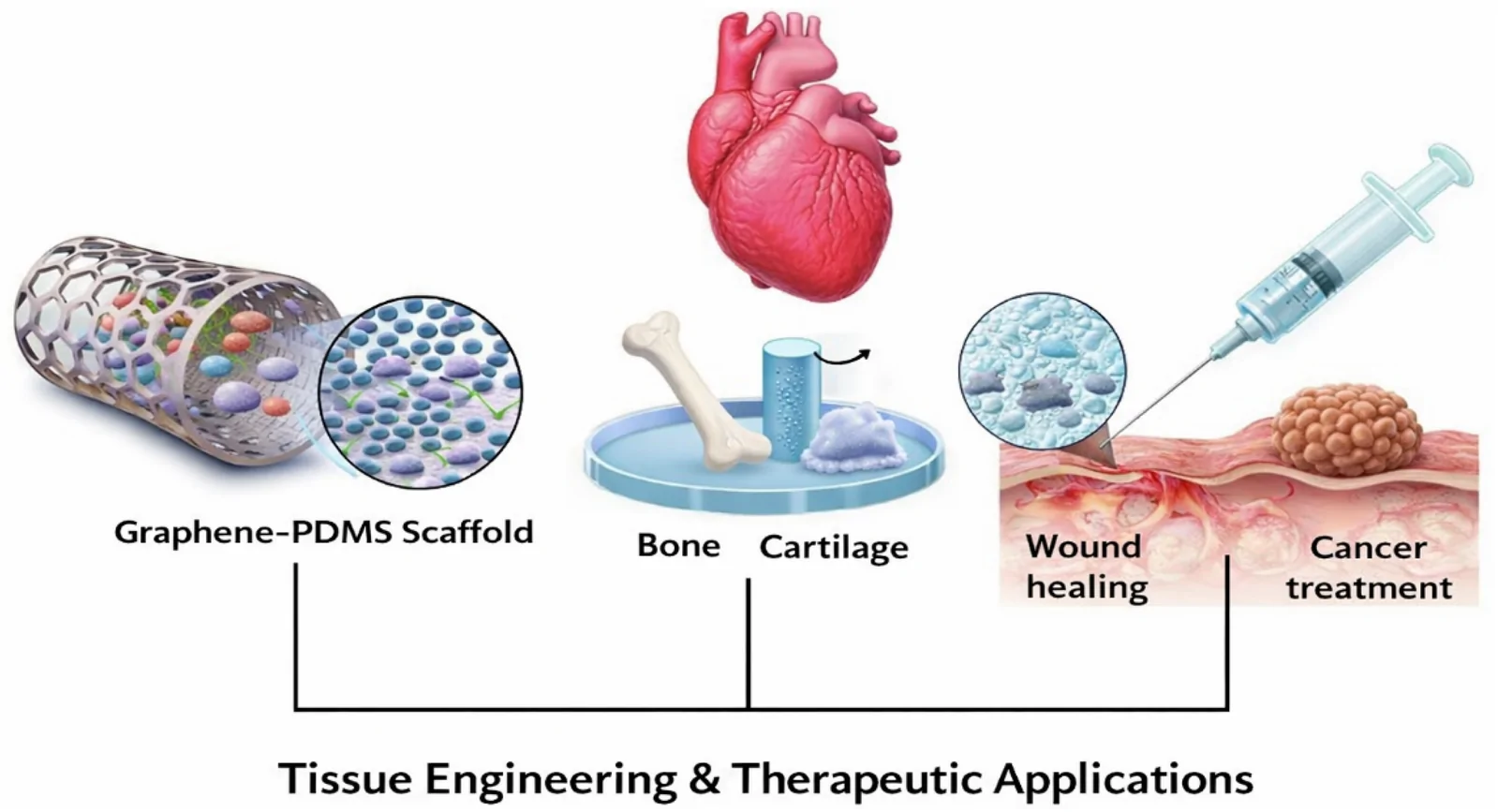
Applications of graphene scaffolds in tissue engineering and therapeutic platforms (neural, cardiac, osteogenic, and wound-healing contexts).

Graphene and its derivatives have shown great promise in tissue engineering because they can reinforce biomaterial scaffolds and promote favourable cellular responses. When incorporated into 3D-printed, electrospun, or hydrogel-based scaffolds, graphene enhances mechanical strength, electrical conductivity, and nanoscale roughness, all of which contribute to improved cell adhesion, proliferation, and lineage-specific differentiation.^183–185^ In neural tissue engineering, graphene's conductivity supports neurite extension and synaptic maturation, facilitating functional nerve regeneration. In cardiac applications, graphene–polymer scaffolds promote synchronous contraction of cardiomyocytes, an essential feature for engineered heart tissues. Similarly, in musculoskeletal and bone regeneration, GO facilitates mineralisation and osteogenic differentiation of stem cells. Beyond structural reinforcement, graphene exhibits intrinsic therapeutic benefits, including antibacterial activity and wound-healing promotion, making it an attractive multifunctional material for regenerative medicine, Fig. 8.

## 8 Photothermal and photodynamic therapy

Graphene's strong absorption in the near-infrared (NIR) region enables efficient conversion of light into localised heat, making it a powerful photothermal agent for cancer therapy.^87,186,187^ Upon NIR irradiation, graphene-based nanomaterials can selectively ablate tumour cells with minimal damage to surrounding tissues.^87,188,189^ In addition, graphene can serve as a carrier for photosensitizers, enabling synergistic photodynamic therapy (PDT) and facilitating combined chemo-photothermal or chemo-photodynamic strategies. Compared to conventional gold nanoparticles, graphene-based systems exhibit higher drug loading capacity, broader optical absorption spectra, and greater versatility in multimodal treatment designs.^190–193^ These advantages underscore graphene's role as a next-generation platform for minimally invasive cancer therapy.

## 9 Implants and neural interfaces

The mechanical flexibility, electrical conductivity, and biocompatibility of graphene make it highly suitable for biomedical implants and neural interfaces. Recent advances have demonstrated ultrathin graphene-based brain implants capable of mapping neural activity with high resolution, distinguishing between healthy and cancerous tissues, and offering improved biocompatibility compared to traditional metallic electrodes.^194^ Such brain-computer interface (BCI) devices minimise inflammation and enhance signal fidelity, addressing critical limitations of conventional implantable systems. Beyond neural applications, graphene-based coatings on orthopaedic and dental implants have been shown to improve osseointegration and reduce bacterial colonisation. Collectively, these applications highlight the multifunctional role of GNPs in implantable biomedical devices, offering safer, more effective, and less invasive therapeutic solutions. Nonetheless, continued research is necessary to optimise surface modifications, address long-term safety concerns, and establish regulatory pathways for clinical translation.^195^

### 9.1 Physicochemical determinants of graphene toxicity: *in vitro* and *in vivo* evidence

Graphene-based materials (GBMs) do not exhibit a single, uniform toxicity pattern. Their biological effects depend on interconnected physicochemical factors, including sheet size, aspect ratio, oxidation level, surface charge, aggregation, functionalization, protein-corona formation, dose, exposure time, and administration route. These factors influence cellular uptake, intracellular behaviour, biodistribution, and adverse responses, making simple comparisons based only on mass concentration insufficient. Lateral size and geometry are major determinants of toxicity. Smaller GO sheets are more readily internalised and may promote intracellular oxidative stress, while larger sheets can interact strongly with cell membranes or trigger frustrated phagocytic and inflammatory responses. Therefore, graphene toxicity should be interpreted using full size-distribution data rather than mean particle size alone.^98,196^

Oxidation state, surface chemistry, charge, and aggregation further shape biological responses. GO is generally more dispersible than pristine or reduced graphene, whereas reduction can increase hydrophobic interactions and aggregation. Functional coatings such as PEG, polysaccharides, or proteins often improve stability and reduce nonspecific membrane interactions, although toxicity depends on the combined effects of dispersion, surface reactivity, defect density, and biomolecular interactions.^197,198^ Aggregation and the protein corona can substantially alter the effective exposure. Ionic conditions and divalent cations may increase aggregation, changing sedimentation, delivered cellular dose, and available reactive surface area. In biological fluids, adsorbed proteins create a new biological identity that can either mask reactive surfaces or promote uptake and immune recognition. Consequently, serum-free experiments may overestimate direct graphene–membrane damage compared with protein-containing environments.^199,200^

Dose, exposure duration, and experimental model are also critical. *In vitro* studies often show limited effects at low concentrations but increased oxidative stress, mitochondrial dysfunction, membrane injury, inflammation, and reduced viability at higher concentrations or longer exposures.^53,201^ However, differences in cell type, serum content, material properties, dispersion methods, and assay design make it difficult to define a universal safe or toxic threshold. *In vivo* toxicity depends strongly on administered dose and exposure route. Animal studies report material accumulation mainly in the liver, spleen, and lungs after systemic exposure, with biodistribution influenced by size, aggregation, and surface modification. Higher intravenous GO doses have been associated with pulmonary accumulation and pathological effects, whereas lower doses and functionalized formulations are generally better tolerated.^14,202^

Generally, GBM toxicity is context-dependent and cannot be predicted from concentration alone. Meaningful comparisons require standardised reporting of material dimensions, oxidation state, surface chemistry, charge, aggregation, corona composition, dose, exposure time, and route of administration. Evidence from human inhalation exposure to thin GO nanosheets suggests no overt short-term pulmonary or cardiovascular harm under the tested conditions, but this does not establish chronic safety or directly translate to high-dose cell-culture or intravenous animal models.^203^ Exposure route further changes the toxicological profile. Intravenous administration provides direct systemic exposure and commonly results in liver, spleen, and/or lung accumulation depending on formulation. Pulmonary exposure emphasises interactions with the respiratory epithelium, alveolar macrophages, and inflammatory pathways, whereas oral administration introduces gastrointestinal barriers and generally produces different systemic bioavailability. Importantly, the first controlled human inhalation study of thin GO nanosheets exposed healthy participants to 200 µg m^−3^ for 2 h and reported no overt acute detrimental effects on pulmonary or cardiovascular function, although the study was designed to assess short-term exposure rather than chronic toxicity or therapeutic safety.^81^ This human exposure result therefore cannot be directly compared with high-concentration cell-culture experiments or intravenous animal studies, Table 5.

**Table 5:** Key physicochemical and exposure-related determinants of graphene-based material toxicity

Factor	Main biological effect	Evidence/exposure	Ref.
Size/aspect ratio	Smaller GO favours uptake; larger sheets can enhance membrane/phagocytic interactions and inflammation	Mainly *in vitro*	98 and 196
Oxidation/surface chemistry	Controls dispersion, membrane interactions, ROS generation and cytotoxicity	Mainly *in vitro*	196 and 204
Aggregation & protein corona	Alters effective cellular dose, uptake, immune recognition and toxicity	*In vitro*/mechanistic	199 and 205
Dose & exposure time	Toxicity generally increases with concentration and prolonged exposure; responses remain material- and cell-dependent	*In vitro*: commonly µg mL^−1^; animal: mg kg^−1^	204
Exposure route & biodistribution	IV, pulmonary and oral exposure produce different organ distributions and toxicological profiles	Animal/*in vivo* human exposure data	14 and 206

### 9.2 The graphene nanostructures in antimicrobial activities

**Fig. 9:**
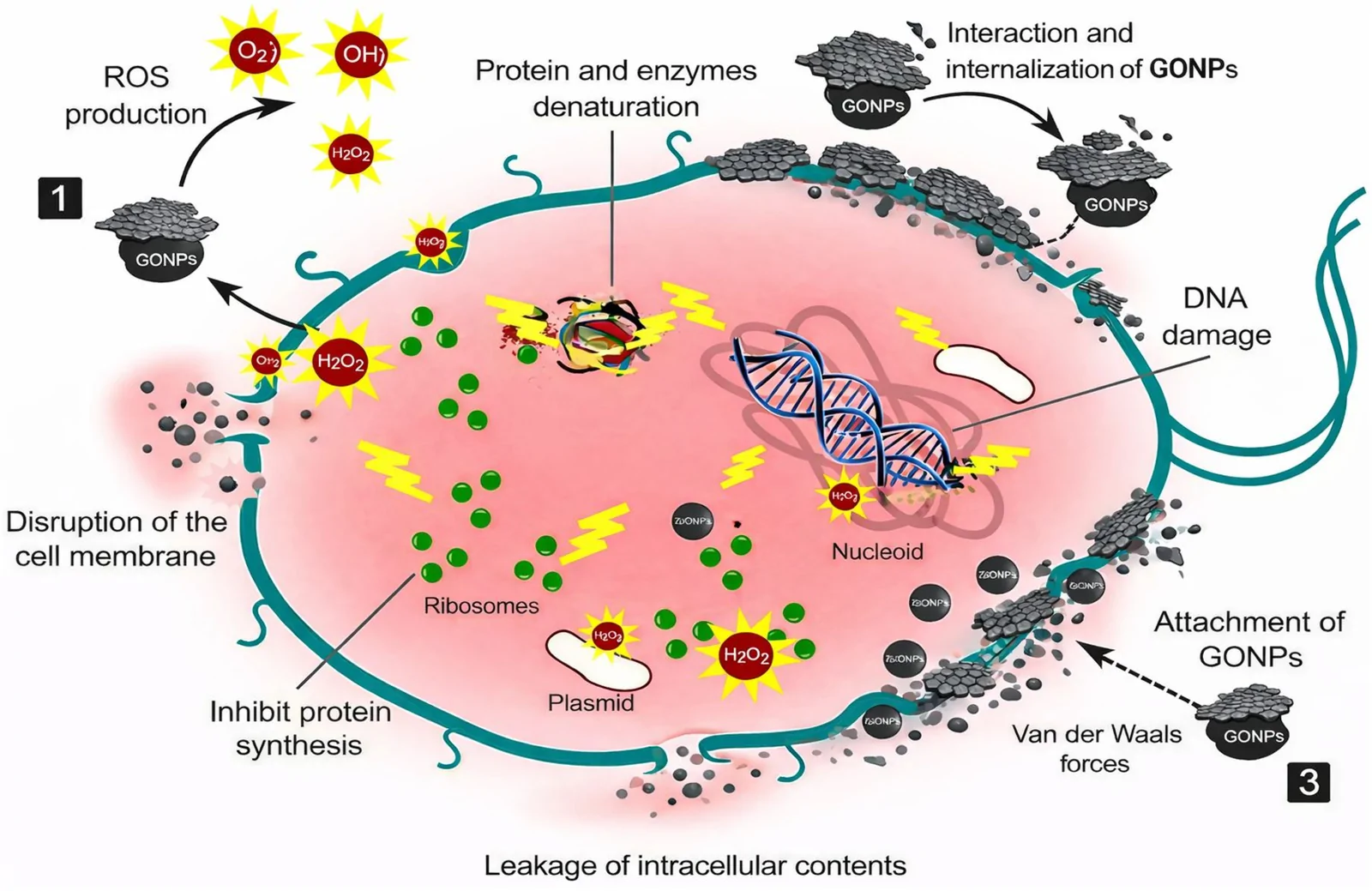
Schematic illustration of primary antimicrobial mechanisms: membrane disruption, ROS-induced oxidative stress, and cell entrapment.

Graphene oxide (GO) is an auspicious material for the development of antimicrobial surfaces due to its contact-based mechanisms. It exhibits broad-spectrum antimicrobial activity and can also serve as a carrier for conventional antibiotics, such as double antibiotic paste (DAP). The combined system (GO-DAP) has demonstrated enhanced antibacterial effects against resilient pathogens like *Enterococcus faecalis*.^207–211^ Despite its potential, the relationship between GO's physicochemical properties and antimicrobial efficacy remains critical. In this context, we investigated the size-dependent activity of GO using *Escherichia coli* as a model Gram-negative bacterium. GO suspensions with sheet areas ranging from 0.01 to 0.65 µm^2^ were evaluated both in suspension and as model surface coatings. For surface coatings, antimicrobial activity increased approximately fourfold as the sheet area decreased from 0.65 to 0.01 µm^2^. This enhanced effect of smaller sheets is attributed to their higher defect density, which facilitates oxidative stress *via* reactive oxygen species (ROS). In suspension assays, the dominant mechanism shifted to cell entrapment, where larger sheets displayed greater antimicrobial effect due to their ability to physically isolate bacterial cells. However, this inactivation was reversible, as cells regained viability when separated from GO sheets by sonication, indicating a bacteriostatic rather than bactericidal mechanism.^116,207^ These findings highlight the importance of considering GO sheet size and application mode when designing antimicrobial surfaces. Smaller sheets are optimal for coatings relying on oxidative mechanisms, whereas larger sheets may be effective in suspension-based applications where physical entrapment is desired. Overall, careful assessment of the structure–activity relationship is essential for developing efficient graphene-based antimicrobial materials,^212^Fig. 9.

## 10 Size-dependent antimicrobial activity of graphene nanoparticles

**Fig. 10:**
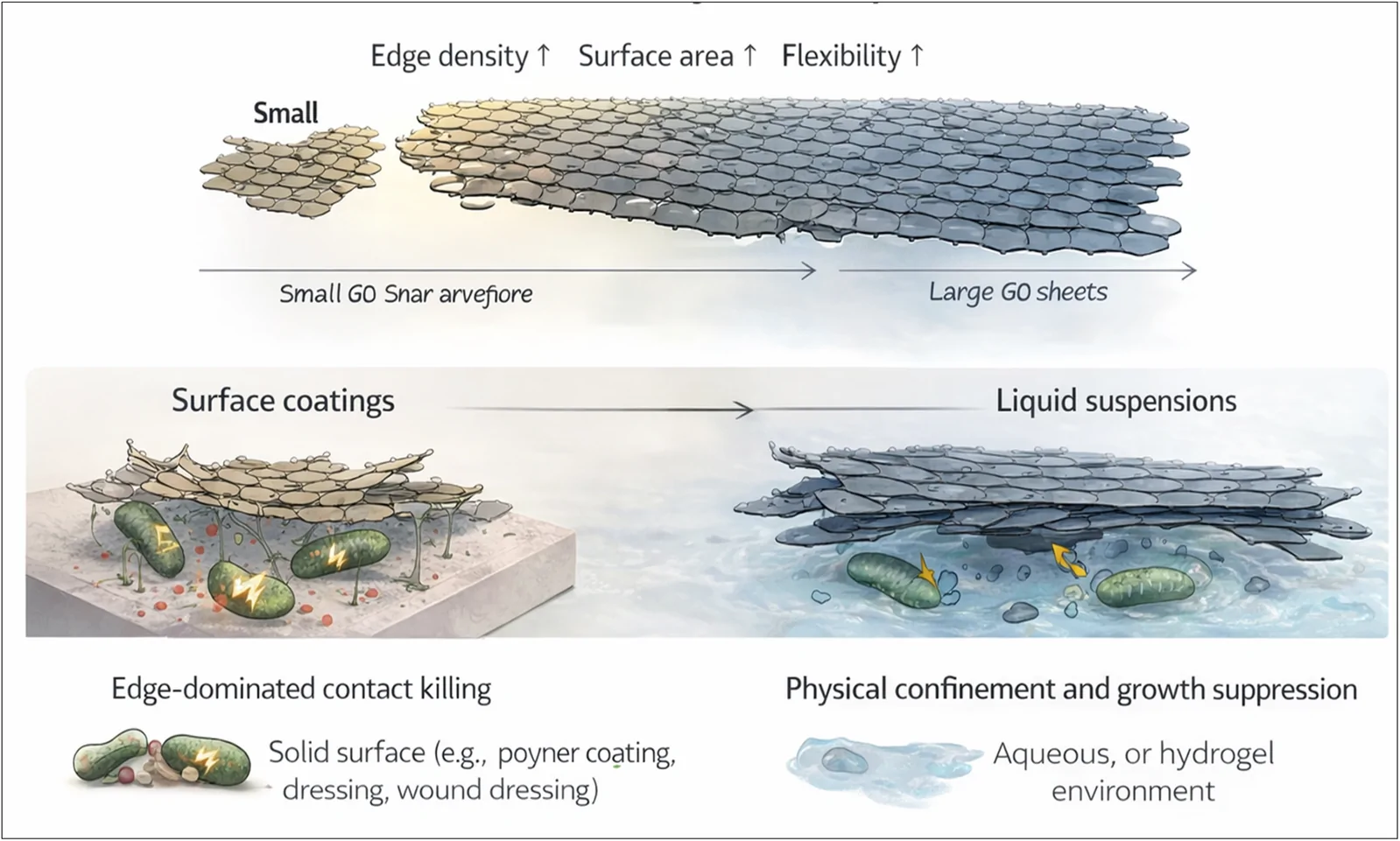
Size-dependent antimicrobial activity: small sheets are more effective in surface coatings, while large sheets enhance cell entrapment in suspensions.

The antimicrobial activity of graphene nanoparticles (GNPs) is strongly influenced by their lateral size, which governs their interactions with microbial cells and determines their mechanism of action.^116,213–216^ Smaller graphene sheets exhibit higher edge density and sharper boundaries, enabling them to physically disrupt bacterial cell membranes through direct contact, often referred to as the “nano-knife” effect. This makes them particularly effective in surface coating applications, where intimate contact with microbial cells enhances antibacterial performance. In contrast, larger graphene sheets possess a greater surface area that facilitates the wrapping and entrapment of bacterial cells, limiting nutrient transport and leading to cell death, especially in suspension systems.^217–221^ Additionally, size-dependent behaviour influences oxidative stress generation and electron transfer processes, further contributing to antimicrobial efficiency. Therefore, controlling the size of GNPs is a critical design parameter for optimising their performance in different biomedical and environmental applications,^70,222–224^Fig. 10.

### 10.1 Antimicrobial mechanisms and safety considerations of graphene-based materials

Graphene-family nanomaterials do not act through one antimicrobial pathway alone.^225,226^ Their activity usually results from several interconnected effects, including physical damage to bacterial membranes, oxidative stress, electron-transfer reactions, microbial wrapping or entrapment, direct oxidative injury, and the release of active functional species. The extent to which each mechanism contributes depends on the graphene derivative, sheet size, oxidation level, aggregation behaviour, surface modification, substrate, bacterial species, exposure duration, and applied concentration. One of the main antibacterial mechanisms is direct contact with the bacterial envelope. Because graphene and GO are composed of extremely thin sheets with sharp edges, they can interact with, deform, or pierce bacterial membranes.^116,227^ This damage may increase membrane permeability and lead to leakage of intracellular proteins, nucleic acids, electrolytes, and phospholipids. Perreault *et al.* reported progressive membrane injury in *Escherichia coli* and *Enterococcus faecalis* following GO treatment, together with greater protein and adenine release as concentration and exposure time increased.^207^ Their reported MIC values were approximately 1 µg mL^−1^ for *E. coli* and 4 µg mL^−1^ for *E. faecalis*, although higher GO concentrations of 50–150 µg mL^−1^ were used for membrane-damage experiments. Previous studies showed that graphene nanosheets can remove phospholipids from bacterial membranes through strong interactions between the graphitic surface and lipid molecules, explaining membrane destabilisation and cell death.^219,228^ Lateral sheet size plays an important role in the antimicrobial behaviour of graphene materials, although its effect depends greatly on the material's physical form and dispersion state. Liu *et al.* reported that GO antibacterial activity increased about fourfold when the average sheet area was reduced from approximately 0.65 to 0.01 µm^2^, suggesting that smaller GO sheets provide more exposed active edges and more efficient membrane contact in surface-associated systems.^227^ However, smaller sheets should not be assumed to be more effective in all cases. Larger, flexible GO sheets can contact multiple parts of a bacterial cell at the same time and may wrap or entrap entire cells, especially in suspension. Akhavan and Ghaderi showed that graphene-family nanosheets can physically enclose bacteria and separate them from their surrounding environment.^218^ Therefore, small sheets are more likely to promote edge-driven membrane disruption, whereas larger sheets may contribute more to bacterial wrapping, entrapment, or physical isolation, depending on dispersion conditions and substrate configuration. Another key antimicrobial pathway is oxidative stress. GO and rGO can disrupt bacterial redox balance and stimulate the generation of reactive oxygen species (ROS), such as superoxide and other reactive intermediates. These ROS may oxidise membrane lipids, proteins, and nucleic acids, leading to membrane damage and reduced metabolic function. O. Akhavan showed that oxidative stress plays an important role in the antibacterial activity of graphene derivatives and that oxidative damage and membrane disruption usually occur together rather than as separate processes.^229^ Graphene-based materials (GBMs) exhibit antibacterial activity through several interrelated physical and chemical mechanisms. Oxidative stress is often involved, as GO can promote ROS generation, lipid peroxidation, and cellular damage. However, this activity is not always fully ROS-dependent; graphene surfaces may also cause ROS-independent oxidative stress by accepting electrons from bacterial cellular components, depleting intracellular reducing equivalents, and disrupting redox homeostasis. Electron transfer is especially important when graphene or reduced graphene oxide (rGO) is placed on conductive or semiconductive substrates, where the restored π-conjugated network can withdraw electrons from bacterial respiratory components and disturb membrane-associated electron transport. Substrate effects should therefore be considered: graphene films on Cu and Ge showed stronger antibacterial activity than those on SiO_2_, indicating that conductivity, electronic properties, and surface chemistry of the support can influence the observed activity. Thus, graphene-coating studies should separate intrinsic graphene effects from antibacterial contributions of the underlying substrate.^230,231^

This distinction is also critical for functionalized graphene nanocomposites. When graphene is combined with Ag, Cu, ZnO, TiO_2_, antibiotics, antimicrobial peptides, or other active agents, the material should be viewed as a multimodal antimicrobial system.^232^ Its antibacterial performance may arise from graphene-mediated membrane disruption and electron-transfer effects, together with released or generated antimicrobial species such as Ag^+^, Cu^2+^, Zn^2+^, nitric oxide, antibiotics, or photocatalytically produced ROS.^232^ Therefore, enhanced activity in graphene–metal or graphene–metal oxide composites should not be attributed solely to graphene, especially when synergistic effects are possible.

Another mechanism is bacterial wrapping and entrapment. Flexible GO sheets can adhere to and surround bacterial cells, isolating them from the surrounding environment and limiting nutrient transport and molecular exchange.^233–235^ Surface chemistry affects this process: highly oxidised, flexible GO generally shows greater wrapping ability, while more reduced and rigid graphene forms may interact differently with bacterial membranes. Importantly, wrapping may suppress bacterial growth without immediate membrane rupture or irreversible cell death, so physical isolation should be distinguished from direct membrane-mediated killing.^233–235^ This relates to the difference between bacteriostatic and bactericidal activity. Bacteriostatic effects inhibit growth or replication without necessarily killing cells, whereas bactericidal activity causes loss of bacterial viability.^236,237^ Therefore, reduced optical density, delayed growth, or inhibition zones alone are insufficient to prove bacterial killing. Bactericidal claims should be supported by viable colony counts, MBC values, membrane-integrity assays, live/dead staining, or evidence of irreversible intracellular leakage.^237^ Graphene antimicrobial studies should therefore distinguish MIC-based growth inhibition from MBC- or viability-based killing. Antimicrobial efficacy should also be assessed alongside mammalian-cell biocompatibility, because graphene toxicity depends on concentration, exposure time, sheet size, aggregation, oxidation degree, surface functionalization, and cell type.^232,238,239^ Graphene nanoplatelets, for example, showed increased mammalian-cell cytotoxicity above about 50 µg mL^−1^, with toxicity rising over time.^239^ Similarly, GO exposure in human ocular epithelial cells at 12.5–100 µg mL^−1^ produced time-dependent effects, with prolonged exposure linked to increased intracellular ROS and cytotoxicity.^238^ These findings show that strong antimicrobial performance does not automatically indicate biological safety. For biomedical use, GBMs should demonstrate an antimicrobial selectivity window, meaning bacteria are inhibited or killed at concentrations that maintain acceptable mammalian-cell viability and function. This is particularly important for dispersed graphene formulations, where cellular exposure may exceed that of immobilised coatings. Future studies should report MIC and MBC values together with mammalian-cell viability, exposure duration, sheet dimensions, aggregation behaviour, oxidation state, and surface chemistry under comparable conditions.^232,238,239^ Such reporting would allow a more reliable evaluation of the balance between antimicrobial efficacy and biological safety.

### 10.2 Sustainability of conventional and green graphene synthesis

The sustainability of graphene-based materials must be evaluated across the full production chain, including synthesis, purification, reduction, and waste treatment rather than inferred from the perceived “greenness” of individual reagents. Hummers-type routes remain common for graphene oxide (GO) production because they offer high yield, reproducibility, and scalability; however, they also rely on concentrated H_2_SO_4_, strong oxidants such as KMnO_4_, intensive washing, and hazardous effluent treatment. Consequently, these methods can involve substantial chemical use, water demand, energy consumption, and waste generation. Further reduction of GO to reduced graphene oxide (rGO) may increase these burdens, particularly when hydrazine, HI, NaBH_4_, or organic solvents are used.^240,241^

Life-cycle assessment (LCA) is therefore essential for quantifying these trade-offs. For example, a modified Hummers route required approximately 25 kJ g^−1^ and produced 1.78 g CO_2_-eq g^−1^ of GO, while subsequent HI/acetic acid reduction increased the values to about 61 kJ g^−1^ and 4.24 g CO_2_-eq g^−1^ of rGO.^242^ Reported carbon footprints for Hummers-type GO also vary widely, from approximately 0.35 to 6.90 g CO_2_-eq g^−1^, depending on yield, reagent consumption, electricity use, purification intensity, and waste-management practices.^241,242^

To reduce reliance on hazardous chemicals, plant extracts, natural polymers, microorganisms, and other biological materials have been explored as alternative reducing agents.^243,244^ These routes may enable aqueous processing under milder conditions and lower toxic-chemical use. Nevertheless, bio-derived processes should not automatically be considered environmentally superior, because feedstock preparation, heating, long reaction times, purification, repeated washing, and variability in biological materials can introduce additional impacts. Their benefits should therefore be demonstrated through quantitative assessment, not assumed from the biological origin of the reducing agent.^244^

Some bio-based routes show promising LCA results. *E. coli*-mediated graphene reduction reportedly consumed about 5 Wh, compared with 1642 Wh for the evaluated chemical reduction process, and reduced global-warming potential by approximately 71%.^245^ Likewise, a cradle-to-gate study reported global-warming potentials of 115.86 kg CO_2_-eq kg^−1^ for graphene derived from *Populus* wood waste and 4841 kg CO_2_-eq kg^−1^ for the evaluated Hummers-derived pathway.^246^ However, because electricity use accounted for about 83% of the biomass pathway's climate impact, bio-derived graphene production remains strongly dependent on energy source and processing conditions.^246^

Scalability is another critical factor. Conventional oxidation methods are generally more reproducible and easier to standardize, but they require strict control of corrosive reagents, exothermic reactions, large washing volumes, and hazardous effluents.^241,242^ Biological and plant-mediated approaches may reduce chemical hazards, yet their scale-up can be constrained by longer processing times, feedstock variability, inconsistent reduction efficiency, and limited batch-to-batch reproducibility.^243–245^ Electrochemical methods can reduce dependence on strong oxidants, but their environmental performance depends on electricity source, electrode lifetime, electrolyte recovery, yield, and purification requirements.^247^ Overall, the green synthesis should be used cautiously and supported by mass and energy balances, waste accounting, scalability analysis, and comparative LCA. Bio-inspired routes are best described as potentially more sustainable alternatives until their advantages over conventional methods are verified quantitatively,^241–247^Table 6.

**Table 6:** Sustainability comparison of conventional and green synthesis routes for graphene-based materials

Route	Key conditions	Quantitative/environmental evidence	Main limitation	Ref.
Hummers/modified Hummers GO	H_2_SO_4_/KMnO_4_; extensive washing	∼25 kJ g^−1^; 1.78 g CO_2_-eq g^−1^	Corrosive reagents, wastewater, high water use	241 and 242
Chemical reduction to rGO	Hydrazine, HI/AcOH, NaBH_4_	∼61 kJ g^−1^; 4.24 g CO_2_-eq g^−1^ (HI/AcOH)	Toxic reagents, purification and waste	241 and 242
Plant-extract reduction	Aqueous plant extracts; moderate temperature	Comprehensive LCA data remain limited	Feedstock variability and reproducibility	243 and 244
Microbial reduction	*E. coli* or other microorganisms	∼5 *vs.* 1642 Wh; ∼71% lower GWP than chemical reduction	Longer processing; upstream GO impacts remain	
Biomass-derived graphene	Biomass pyrolysis/activation	∼115.9 *vs.* 4841 kg CO_2_-eq kg^−1^ for compared Hummers route	Pyrolysis energy and activation chemicals	246
Natural-polymer/bio-inspired	Chitosan, cellulose, proteins	Limited quantitative comparative LCA data	Batch variability, purification and reproducibility	243 and 244
Electrochemical synthesis	Electrochemical oxidation/exfoliation	Lower impacts reported in several environmental categories	Electricity and electrode/electrolyte impacts	247

## 11 Challenges and future targets for GNPs applications

Despite their remarkable promise in drug delivery, biosensing, tissue engineering, and antimicrobial applications, the clinical translation of graphene nanostructures (GNPs) remains constrained by several key challenges. A paramount concern is the incomplete understanding of their long-term toxicity and biocompatibility.^42,50,136,203^ Reported findings show significant variation depending on particle size, oxidation state, and surface functionalization. The absence of standardised toxicological assays further complicates risk assessment, underscoring an urgent need for harmonised evaluation frameworks. Reproducibility presents another significant barrier. Inconsistent synthesis methods for graphene oxide (GO), reduced graphene oxide (rGO), and graphene quantum dots (GQDs) often result in heterogeneous materials with divergent physicochemical properties.^136,248,249^ These inconsistencies lead to unpredictable biological performance and hinder scalable production, underscoring the necessity for standardised synthesis protocols and rigorous characterization. From a regulatory standpoint, no graphene-based therapeutic has yet received FDA approval. Complex biological interactions, uncertainties in biodistribution, and potential environmental impacts pose major hurdles. Addressing these issues requires the integration of comprehensive toxicological, pharmacokinetic, and efficacy data into clear regulatory guidelines. Sustainability also emerges as a critical challenge. Conventional synthesis routes frequently involve harsh chemicals and energy-intensive processes, raising ecological concerns. While green synthesis approaches utilising plant extracts, natural polymers, and bio-inspired reductants offer promising alternatives, they require further optimisation to achieve scalability and cost-effectiveness.^143^ Future research should prioritise developing reproducible and sustainable synthesis techniques, advancing precise surface functionalization to enhance targeting and safety, and designing multifunctional theranostic platforms that integrate diagnosis and therapy. Establishing standardised toxicity protocols and conducting long-term *in vivo* studies will be crucial to bridging the gap between laboratory innovation and clinical application. These efforts are essential to establishing graphene nanostructures as viable and sustainable pillars of next-generation nanomedicine.

## 12 Conclusion

Graphene nanostructures represent a groundbreaking class of biomaterials with significant implications for next-generation medicine. Their unique physicochemical properties enable versatile applications in drug delivery, biosensing, tissue regeneration, neural engineering, and antimicrobial coatings. However, unresolved issues related to reproducibility, long-term safety, and regulatory approval remain critical bottlenecks. Continued research into eco-friendly synthesis, advanced functionalization, and standardised toxicological assessment will be key to bridging laboratory innovation and clinical practice.

## Author contributions

Sameer Awad: review & editing, writing – original draft, resources, supervision. Ahmed Al-Kubiasi: writing – review & editing, writing, resources. Numan AlHeety: writing – review & editing, writing. Eman Khalaf: review & editing, writing. Doaa A. Elsayed: review & editing, writing.

## Conflicts of interest

The authors declare that the research was conducted in the absence of any commercial or financial relationships that could be construed as a potential conflicts of interest.

## Data Availability

No new data were generated or analyzed in support of this research.
